# COVID-19 Disparities Among Marshallese Pacific Islanders

**DOI:** 10.5888/pcd18.200407

**Published:** 2021-01-07

**Authors:** Pearl A. McElfish, Rachel Purvis, Don E. Willis, Sheldon Riklon

**Affiliations:** 1 College of Medicine, University of Arkansas for Medical Sciences Northwest, Fayetteville, Arkansas; 2 Office of Community Health and Research, University of Arkansas for Medical Sciences Northwest, Fayetteville, Arkansas

Rates of coronavirus disease 2019 (COVID-19) infections, hospitalizations, and deaths differ across racial and ethnic groups in the United States and disproportionately affect minority communities (1–6). In states reporting disaggregated data for Pacific Islander populations, rates of COVID-19 infections, hospitalizations, and deaths were significantly higher among Pacific Islanders than among the White population and other racial and ethnic minority populations (4,7,8). Because the disparities were so alarming among Marshallese Pacific Islanders in northwest Arkansas (ie, Benton and Washington counties), the Centers for Disease Control and Prevention (CDC) conducted a multi-week site visit in late June and early July 2020. Their July 2020 report details the disproportionate effect of COVID-19 on the Marshallese population in this region (9). Marshallese are estimated to make up just 1.5% to 3% of the total population of Benton and Washington counties, yet they accounted for 19% of all COVID-19 cases in the region (9). Of Marshallese patients with COVID-19 during March–June 2020, 9% were hospitalized (9). Nationally, CDC reported that 144.1 per 100,000 (<0.01%) of patients with COVID-19 were hospitalized as of June 2020. Most alarmingly, Marshallese people accounted for 38% of the reported deaths in the 2-county region during March–June 2020 (9). The Marshallese community in northwest Arkansas bears a much larger burden of COVID-19 infection, hospitalization, and death than other racial and ethnic minorities (2,9).

Marshallese people are from the Republic of the Marshall Islands (RMI), a chain of 5 volcanic islands and 29 coral atolls situated in the central Pacific Ocean halfway between Hawaii and Australia (10). From 1946 to 1958, the US military established a nuclear weapons testing program in the RMI and conducted 67 nuclear tests (11). These tests were equivalent in payload to 7,200 Hiroshima-size bombs (11). The nuclear tests caused acute radiation exposure and serious long-term health effects for the Marshallese community (12,13). The nuclear tests also devastated the environment, and sources of food (eg, fish, breadfruit, coconut) were contaminated, which resulted in the adoption of a diet high in fat and processed commodity food, which negatively affected the health of these Pacific Islanders (13–15). A Compact of Free Association Agreement (COFA) between the RMI and the United States was signed in 1986; this agreement granted the US exclusive control of a strategic base of operation for the US military and gave the Marshallese the right to visit, reside, work, and study in the United States without a visa (16). Although COFA migrants are not US citizens, they do pay taxes and serve in the US military at greater per capita rates than US citizens (17). The Marshallese diaspora is driven by a lack of economic and employment opportunities in the RMI and the desire for greater access to education, employment, and health care in the United States (18). Marshallese migration to Springdale, Arkansas, began with John Moody, who found work with Tyson Foods after attending college in eastern Oklahoma. Moody’s description of the low cost of living and employment opportunities initiated the first wave of Marshallese migration to northwest Arkansas in the 1980s, and Springdale is now home to the largest Marshallese community in the continental United States (19,20).

Chronic diseases increase the risk of complications and death from COVID-19 (21,22). Marshallese living in the United States have a high prevalence of cardiometabolic comorbidities, including type 2 diabetes, which is known to increase the risk of severe infection and mortality from COVID-19 (21,22). In 2015, the prevalence of type 2 diabetes among Marshallese adults was 38.4%, and the prevalence of prediabetes was 32.6% (23); at the same time the prevalence of diabetes in 2015 among the general US adult population was 13% (24). In addition, Marshallese have high rates of infectious diseases, such as tuberculosis and Hansen’s disease (leprosy) (25,26). A local needs assessments demonstrated in 2015 that 49.6% of Marshallese adults had not seen a physician in the previous year because of cost (23). Even after the passage of the Affordable Care Act and the expansion of Medicaid, the proportion of uninsured Marshallese adults in a local needs assessment was 48%, more than 5 times higher than the proportion of uninsured adults at the national (8.0%) and state (9.1%) levels (27). This disparity is most likely due to the lack of Medicaid eligibility for COFA migrants (8,17). Although socioeconomic data on Marshallese are sparse, the available literature shows that many Marshallese community members face socioeconomic challenges, such as low educational attainment, unstable and dense housing, and low-wage jobs. Many low-wage jobs are in the poultry industry or are essential jobs that do not allow working from home (9,28).

Strategies to address COVID-19 among Marshallese must consider the historic trauma caused by nuclear testing among this population (15,18,29–31). After nuclear testing in the RMI, US scientists experimented on Marshall Islanders who were exposed to nuclear fallout. Marshallese were interned in a camp to study the effects of radiation injuries on humans (11). The research was conducted without the informed consent of participants and without translation of study information into the native language (11). Like other people marked by historic trauma, Marshallese distrust health care providers and researchers (32). Public health research in other areas (eg, diabetes, prenatal care) among the Marshallese population documented barriers at individual, interpersonal, organizational, community, and policy levels (33) and showed that stigma may inhibit help-seeking behaviors (34).

Our study team is overcoming this distrust by using a community-based participatory research (CBPR) approach. CBPR shares power and builds trust between academic researchers and community partners (35–40). The University of Arkansas for Medical Sciences (UAMS) has engaged in CBPR with the Marshallese community since 2013 to address health disparities. CBPR engages community partners and honors their unique contributions to integrate and leverage contextually and culturally situated knowledge, practices, and resources. During 2013–2020, UAMS worked with the Marshallese community on 8 locally and federally funded projects to address health disparities and collaborated to launch a clinic providing free health care to the Marshallese (23,41–46). The CBPR team has implemented culturally tailored diabetes self-management education and diabetes prevention programs and worked to improve access to healthy food to prevent chronic disease (44,47,48). The team includes more than 20 Marshallese staff members working at the university and community-based organizations, Marshallese nurses, and a Marshallese physician. It is led by a community advisory board and include subcontracts to several nonprofit organizations led by Marshallese people (44,47,49,50). The partnership was funded by a CDC program, Racial and Ethnic Approaches to Community Health (REACH). The REACH program uses community-engaged approaches and is focused on supporting culturally tailored interventions to address preventable health conditions, linking community and clinical efforts to increase access to health care and preventive care programs at the community level, and implementing, evaluating and disseminating practice- and evidence-based strategies to reduce health disparities in chronic conditions (51).

In March 2020, we began leveraging our REACH partnership to address COVID-19 in the Marshallese community. The objective of this essay is to describe the COVID-19 Comprehensive Response Plan for the Marshallese Community in northwest Arkansas. Although no REACH funding is spent on COVID-19 activities, the community-engaged capacity developed through REACH implementation has provided a strong foundation for the COVID-19 Comprehensive Response Plan.

## A COVID-19 Comprehensive Response Plan for the Marshallese Community in Northwest Arkansas

The CBPR partnership comprises UAMS; Community Clinic, a federally qualified health center; the Arkansas Department of Health; 4 local hospitals (Mercy, Arkansas Children’s Hospital, Washington Regional Medical Center, Northwest Health); the Marshallese Consulate in Springdale, Arkansas; Marshallese Educational Initiative; Arkansas Coalition of Marshallese; Faith in Action Research and Resources Alliance; Kili Bikini Ejit; Northwest Arkansas Council (a local convening and economic development organization); and local government officials, employers (including the poultry industry), and nonprofit organizations. Members have met at least twice per week since March 2020 with daily communication between partners.

The CBPR partnership developed a COVID-19 Comprehensive Response Plan with 4 interrelated components to ensure coordinated effort for Marshallese testing, contact tracing, enhanced case management, and health education.

### The 4 components of the response plan

**Component 1: A collaborative approach to testing.** The COVID-19 Comprehensive Response Plan facilitates testing in a sustainable and distributed manner that leverages the current resources of Community Clinic, the Arkansas Department of Health, and local health care providers and enhances testing efforts through culturally and linguistically appropriate navigation of testing for Marshallese community members. Testing focuses on symptomatic people, nonsymptomatic people who have had contact with a person who is infected or suspected of being infected, and hot spots within organizations or in neighborhoods where multiple COVID-19 cases have been identified. Contacts are offered the option of being tested in 1 of 3 testing locations in the community or being tested in their home by a nurse-led testing team. Staff members bilingual in English and Marshallese are part of the testing teams for every home-based and community-based testing. The plan also provides a bilingual screening hotline.

**Component 2: A dedicated contact tracing center.** The COVID-19 Comprehensive Response Plan includes a dedicated contact tracing center in northwest Arkansas that fully coordinates with the Arkansas Department of Health. The contact tracing center uses the same software, policies, and procedures as the Arkansas Department of Health to identify and follow up with all people who may have come into contact with a person infected with COVID-19. The contact tracing center team includes bilingual contact tracing staff members, nurses, and social workers. A positive test result for COVID-19 is a mandatory reportable diagnosis that must be immediately reported to the Arkansas Department of Health. Newly diagnosed people (hereinafter, case patients) whose preferred language is Marshallese are referred by Arkansas Department of Health officials to the Northwest Arkansas Contact Tracing Center for Marshallese. Staff members at the center reach out to case patients and determine who they have been in direct contact with during the period from 2 days before symptoms started until the beginning of quarantine. Case patients are asked to inform all contacts that someone from the contact tracing center will be calling them. Center staff members reach out to all contacts and instruct their households and any other direct contacts about quarantine. All contacts are enrolled in a surveillance system to be tracked for 14 days, with a daily call to all contacts in quarantine. All contact tracing data are entered into the software required by the Arkansas Department of Health. Data are uploaded to the Arkansas Department of Health at the end of each day, following an existing protocol under a memorandum of understanding between UAMS and the department. Contacts who need testing are referred to a testing site, and home-based testing is performed if needed (component 1). Contacts who need assistance with food, housing, or medication work with a social worker and bilingual navigators to identify resources to meet those needs (component 3).

**Component 3: Enhanced case management and supported quarantine.** Marshallese people face socioeconomic challenges that are often exacerbated by a COVID-19 diagnosis. Once a case of COVID-19 is identified, contact tracers reach out to people who may have been exposed to the confirmed case patient to have them self-quarantine for 14 days. It is critical to provide the support services that case patients and their close contacts need to self-quarantine, including the provision of essential items, such as food and medications; coordination with worksites; and coordination with community social and behavioral health services. After the initial contact tracing interview, an enhanced case management process is used to monitor the case patient’s health status for any indications of worsening symptoms and work with existing referral patterns to ensure follow-up. In addition, the plan includes the assessment of the case patient’s or contact’s ability to self-isolate and the provision of resources and referrals for the patient, if needed. Standard contact tracing encourages contacts to stay home and maintain distance from others until 14 days after their last exposure. The enhanced case management team provides additional services: they make follow-up calls to case patients and their contacts to encourage them to remain diligent with isolation; check on the contacts’ health and determine whether they develop symptoms; provide resources, education, information, and connection with health care and community-based support organizations; and arrange for food deliveries and prescription drug refills, if needed. Social workers, nurses, and bilingual navigators make up the enhanced case management team. The enhanced case management is coordinated by UAMS and includes the Community Clinic, the Marshallese Educational Initiative, and the Arkansas Coalition of Marshallese. These organizations have subawards from UAMS and are responsible for a collaboratively defined scope of work. Nonprofit partners have leveraged community donations to provide food and housing assistance to Marshallese people who need support to self-quarantine.

**Component 4: Health education and prevention.** The CBPR partnership has worked together to develop COVID-19 communications to increase prevention, testing, quarantine, and follow-up care among the Marshallese community. Communications are based on our previous CBPR research, and all communications were co-developed with Marshallese community members (23,52,53). From March through July 2020, we launched 11 Facebook live sessions, which garnered approximately 25,000 views each. We launched 18 short YouTube videos that covered topics such as proper mask use, social distancing, and quarantine and isolation guidance. We developed written educational materials focused on COVID-19’s effect on pregnancies and people with diabetes and asthma, along with guidance for funerals, church services, and other common situations where transmission is increased (Figure). We also developed and launched a COVID-19 faith-based tool kit for churches and other faith organizations. All materials are in the Marshallese language and include photos of Marshallese people. All materials are reviewed by an infectious disease physician and a Marshallese physician or Marshallese registered nurse at UAMS prior to publication. Communications are consistent with CDC health recommendations, and the communication and dissemination plan are consistent with recommendations CDC made after their July 2020 site visit. Communications tools can be found at https://northwestcampus.uams.edu/ochrcovid. Educational materials have been shared widely throughout Arkansas, as well as other US states, including Oklahoma, Washington, and Hawaii, where large communities of Marshallese people live. We estimate that we have reached some 50,000 Marshallese community members throughout the United States.

**Figure:**
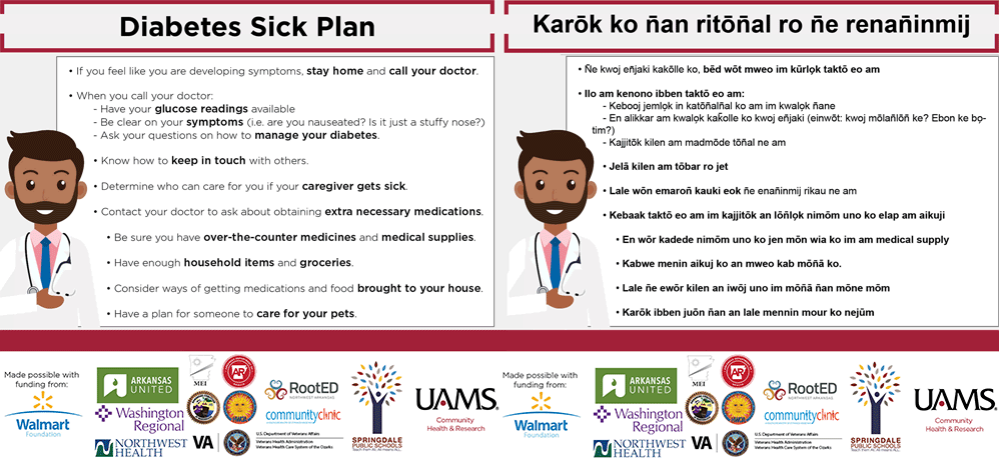
An example of an infographic on a diabetes sick plan during the coronavirus disease 2019 pandemic prepared in both Marshallese and English.

### Staffing

Partial funding has been received from the state of Arkansas through the Coronavirus Aid, Relief and Economic Security (CARES) Act (54) to implement the COVID-19 Comprehensive Response Plan. Most (11 of 15) staff members hired to implement the plan are bilingual in English and Marshallese, and subcontracts from UAMS were provided to partner nonprofit organizations. The bilingual staff members who make up the contact tracing team, navigation team, and testing team speak the Marshallese language fluently. Marshallese staff members have a deep understanding of the Marshallese culture and are trusted members of the community. The additional staff members (nurses, physicians, social workers, and data and information technology staff) who are not Marshallese participate in cultural humility training that is co-led by Marshallese leaders.

### Ongoing CBPR efforts

Partners continue to meet at least twice per week during the implementation of this plan. Co-learning and process improvement is collaboratively achieved through meetings with the implementation team, which includes Marshallese contact tracers, Marshallese navigators, Marshallese nurses, and a Marshallese physician. In addition, weekly meetings of community members are facilitated by the Marshallese Consulate, and monthly meetings are held with all nonprofit organization partners. Many educational materials are developed directly by the community partners, and educational materials developed by UAMS receive extensive input from Marshallese community members. Educational materials include partners’ logos. Partners and community stakeholders are provided updates on process and outcome measures every 2 weeks. This CBPR approach is important to ensuring that efforts reduce stigma and effectively reach the Marshallese community.

### Evaluation

A process and outcome evaluation will be implemented for the COVID-19 Comprehensive Response Plan. Process measures include the number of Marshallese people served with contact tracing, the number and type of enhanced case management services provided, the number and type of testing provided, and the number, type, and reach of health education and prevention efforts. The key outcome measures being examined are the proportion of Marshallese COVID-19 infections, hospitalizations, and deaths in the 2-county region. Lessons learned will also be documented through weekly quality improvement meetings.

### Continued efforts to address chronic disease prevention and control

Although partners have shifted their primary focus to addressing COVID-19, UAMS and community partners continue to address chronic cardiometabolic conditions such as type 2 diabetes. These efforts include primary and secondary prevention of type 2 diabetes through promoting access to healthy food, self-management education, and continued clinic visits during the COVID-19 pandemic. Although the goals for primary and secondary prevention of type 2 diabetes are the same as they were before the pandemic, the methods for implementing them have changed to adhere to CDC recommendations for social distancing. For example, UAMS and partners were providing diabetes prevention program and diabetes self-management in group settings with in-person classes, and we have now shifted to offering group diabetes self-management education classes via interactive video and YouTube videos, rather than in-person classes. These methods will be evaluated for effectiveness. In addition, UAMS and partners is launching a program in early 2021 to test the effectiveness of an intervention that includes the home delivery of diabetes-appropriate food boxes with written diabetes education materials and recipes to aid food-insecure people in managing their type 2 diabetes during COVID-19. With concern that many patients will not attend their regular primary care visits, outreach efforts have focused on ensuring that Marshallese patients understand how to access available telemedicine resources and understand the importance of continuing with health care appointments. UAMS and partners are in the process of launching a pilot program to evaluate the effectiveness of remote patient monitoring paired with telemedicine among Marshallese with uncontrolled type 2 diabetes.

## Conclusion

Marshallese in northwest Arkansas have experienced disproportionate rates of COVID-19 infections, hospitalizations, and deaths. These high rates may be due in part to the high incidence of type 2 diabetes in the Marshallese community, a condition that increases the risk of complications and death from COVID-19 (21,22,55,56). Community-based partners funded through a CDC REACH award have built a strong collaborative foundation to address chronic diseases and associated risk factors in the Marshallese community. We have leveraged that collaboration to address COVID-19 disparities through the development and implementation of a COVID-19 Comprehensive Response Plan based on CDC recommendations (1). Our Comprehensive Response Plan includes increased testing, contact tracing, enhanced case management, and health education. Simultaneously, partners have shifted to remote delivery of health education efforts as we continue to address type 2 diabetes in the Marshallese community. The COVID-19 Comprehensive Response Plan demonstrates how CBPR infrastructure created by the REACH program can be leveraged to reduce health disparities and implement critical CDC recommendations beyond individual grant awards.
